# 

**Published:** 1908-12

**Authors:** 


Sixty-fourth Year.

VOL. LXIV. DECEMBER, 1908. No. 5

### BUFFALO MEDICAL JOURNAL

Established 1845 by AUSTIN FLINT M. D.

[illegible] SURGEON GENERAL'S OFFICE
DEC [illegible]

LIBRARY
Indexed
D. S. G. O.

EDITOR:
WILLIAM WARREN POTTER, M. D.
ASSISTANT EDITORS:
WILLIAM C. KRAUSS, M. D. NELSON W. WILSON, M. D.
ASSOCIATE EDITORS:
JAMES WRIGHT PUTNAM, M. D. ERNEST WENDE, M. D.
JOHN PARMENTER, M. D. JOHN A. MILLER, PH. D.
MAUD J. FRYE, M. D.

